# Telomere Length and Physical Performance at Older Ages: An Individual Participant Meta-Analysis

**DOI:** 10.1371/journal.pone.0069526

**Published:** 2013-07-26

**Authors:** Michael P. Gardner, Carmen Martin-Ruiz, Rachel Cooper, Rebecca Hardy, Avan Aihie Sayer, Cyrus Cooper, Ian J. Deary, John Gallacher, Sarah E. Harris, Paul G. Shiels, John M. Starr, Diana Kuh, Thomas von Zglinicki, Yoav Ben-Shlomo, the Halcyon study team

**Affiliations:** 1 School of Social and Community Medicine, University of Bristol, Canynge Hall, Bristol, United Kingdom; 2 Institute for Ageing and Health, Newcastle University, Newcastle upon Tyne, United Kingdom; 3 MRC Unit for Lifelong Health and Ageing, University College London, London, United Kingdom; 4 Medical Research Council Lifecourse Epidemiology Unit, University of Southampton, Southampton, United Kingdom; 5 Centre for Cognitive Ageing and Cognitive Epidemiology, University of Edinburgh, Edinburgh, United Kingdom; 6 Department of Psychology, University of Edinburgh, Edinburgh, United Kingdom; 7 Department of Primary Care and Public Health, Cardiff University, Cardiff, United Kingdom; 8 Medical Genetics Section, University of Edinburgh, Edinburgh, United Kingdom; 9 Institute of Cancer Sciences, University of Glasgow, Glasgow, United Kingdom; 10 Alzheimer Scotland Dementia Research Centre, University of Edinburgh, Edinburgh, United Kingdom; University of Valencia, Spain

## Abstract

**Background:**

Telomeres are involved in cellular ageing and shorten with increasing age. If telomere length is a valuable biomarker of ageing, then telomere shortening should be associated with worse physical performance, an ageing trait, but evidence for such an association is lacking. The purpose of this study was to examine whether change in telomere length is associated with physical performance.

**Methods:**

Using data from four UK adult cohorts (ages 53–80 years at baseline), we undertook cross-sectional and longitudinal analyses. We analysed each study separately and then used meta-analytic methods to pool the results. Physical performance was measured using walking and chair rise speed, standing balance time and grip strength. Telomere length was measured by quantitative real-time polymerase chain reaction (PCR) in whole blood at baseline and follow-up (time 1, time 2).

**Results:**

Total sample sizes in meta-analyses ranged from 1,217 to 3,707. There was little evidence that telomere length was associated with walking speed, balance or grip strength, though weak associations were seen with chair rise speed and grip strength at baseline (*p = *0.02 and 0.01 respectively). Faster chair rise speed at follow-up, was associated with a smaller decline in telomere length between time 1 and time 2 (standardised coefficient per SD increase 0.061, 95% CI 0.006, 0.115, *p = *0.03) but this was consistent with chance (*p* = 0.08) after further adjustment.

**Conclusions:**

Whereas shortening of leukocyte telomeres might be an important measure of cellular ageing, there is little evidence that it is a strong biomarker for physical performance.

## Introduction

Telomeres are nucleoprotein complexes at chromosome ends, where the DNA component is a repetitive stretch of (TTAGGG), which caps and protects the end of the chromosome. Since telomeres are involved in cellular ageing and shorten with increasing age, it has been proposed that telomere length could be a useful biomarker of ageing [1]. Whereas some studies have shown that shorter telomeres are associated with obesity [2], male gender [3], lower socioeconomic position [4] and current smoking [5], evidence that telomere length is a biomarker of ageing is equivocal [1]. Although some studies have shown that shorter telomeres are associated with increased mortality rates [6], others have not shown an association [7]. In addition, no evidence of an association between telomere length and frailty was found in a study of adults aged 65 years and older even though the frailty index (physical, psychological and functional domains) is supposedly a good measure of biological ageing [8].

Physical performance can be assessed using simple, objective measures such as chair rise time, grip strength, standing balance and walking speed [9]. Furthermore, reduced performance in each of these measures is a predictor of all cause mortality [9]. We are aware of only four studies which have examined the associations between telomere length and objective measures of physical performance [10]–[13]. None of these studies found evidence of an association between telomere length and grip strength [10]–[13]. Furthermore, no evidence of an association was observed between telomere length and walking speed in the two studies which have examined this [11], [12]. A limitation of these four studies is that they are cross-sectional and did not examine chair rise time or standing balance.

We have undertaken an individual participant data (IPD) meta-analysis [14] using four cohorts, including new unpublished data, as part of the Healthy Ageing across the Life course (HALCyon) programme, to assess the associations between telomere length and four physical performance tests. Each cohort has repeat telomere length measures (time 1 and time 2), with the period between times ranging from 7.5 to 10.2 years. Our approach has several advantages: (a) ability to examine whether age-related changes in telomere length are associated with physical performance; (b) greater statistical power to detect modest associations; (c) standardising analyses by handling covariates and physical performance measures in the same way across studies. The aims of this study were to investigate whether longer telomeres were associated with: (1) better physical performance in cross-sectional analyses, (2) better future physical performance in prospective analyses, whether (3) telomere length at baseline predicted change in physical performance and whether (4) change in telomere length was associated with better physical performance at follow-up.

## Methods

### The Cohorts

The Healthy Ageing across the Life Course (HALCyon) programme is a cross-cohort study on ageing. We have included data from four of the nine UK cohort studies involved in HALCyon which have data on both telomere length and physical performance. These were the Caerphilly Prospective Study (CaPS) [15], the Hertfordshire Ageing Study (HAS) [16], the Lothian Birth Cohort 1921 (LBC1921) [17] and the MRC National Survey of Health and Development (NSHD) [18], [19]. (Appendix S1).

### Ethics Statements

Ethical approval was given for the CaPS by the Ethics Committee of the Division of Medicine of the former South Glamorgan Area Health Authority. Approval for phase 5 came from the South East Wales Research Ethics Committee. For HAS, ethical approval was given by the Bedfordshire and Hertfordshire Local Research Ethics Committee and the West Hertfordshire Local Research Ethics Committee. Ethical approval was given for LBC1921 by the Multicentre Research Ethics Committee for Scotland and the Lothian Research Ethics Committee. Ethical approval for NSHD was given by the North Thames Multi-Centre Research Ethics Committee (age 53 y) and the Central Manchester Research Ethics Committee and the Scottish A Research Ethics Committee (age 60–64).

### Data Availability

Data access for CaPS is available via a request to the data steering committee (see http://www.bris.ac.uk/social-community-medicine/projects/caerphilly/). Bona fide researchers can apply to access the HAS data via a standard application procedure (further details available at : http://www.mrc.soton.ac.uk). Bona fide researchers can apply to access the LBC1921 data via a standard application procedure. Further details available in the open accessed article [17]. Bona fide researchers can apply to access the NSHD data via a standard application procedure (further details available at : http://www.nshd.mrc.ac.uk/data.aspx).

### Physical Performance Measures

Different tests were available at baseline and follow-up across the four cohorts (Appendix S2).

#### Walking speed

In CaPS and NSHD, the time to get up from a chair, walk 3 m at normal speed, turn around, walking back and sitting down was recorded (TUG test) [20]. In HAS, participants walked at a normal pace over a 3 m course. In LBC1921 participants walked as quickly but as safely as possible over a 6 m course and the times recorded. In HAS, LBC1921 and NSHD one trial was performed and in CaPS two trials were performed and the average value used.

#### Chair rises

The time taken for participants to stand up from a chair and sit down as fast as possible five times in HAS and ten times in NSHD, was recorded.

#### Grip strength

Dynamometers were used to record either two (NSHD) or three (HAS) measures in each hand or three measures in the dominant hand (LBC1921). In each of the cohorts, maximum grip strength (kg) achieved was used in analyses.

#### Standing balance

In each of the cohorts, standing balance was the longest time up to 30 s that a participant could stand on one leg with eyes open. One trial was performed in HAS and NSHD and two trials were performed in CaPS (with the best value used).

#### Harmonisation of physical performance measures

To take account of the different protocols used in assesssing physical performance across cohorts, we harmonised these measures [21]. Walking times were converted into walking speed (metres/minute) and chair rise times were converted into chair rise speed (stands/minute) and these measures were standardised by computing study-specific z-scores. Balance time was dichotomised with those in the bottom 20^th^ centile classified with poor balance and compared with the rest of the participants.

### Telomere Length Measures

Telomere length was measured by quantitative real-time polymerase chain reaction (PCR) in whole blood DNA as abundance of telomeric template versus a single gene [7]. All measurements were undertaken in the same laboratory. Measurements were performed in quadruplicate on an Applied Biosystems 7900 HT Fast Real Time PCR system with 384-well plate capacity. The intra-assay coefficient of variation was 2.7% while the inter-assay coefficient of variation was 5.1%. To correct for inter-plate variation, four internal control DNA samples of known telomere length were run within each plate and used to generate a regression line by which values of relative telomere length for the actual samples were converted into absolute telomere lengths in base pairs. For details of primer sequences (including telomeric primers and control gene primers) and a description of the DNA isolation methods see Appendix S3.

### Clinical and Questionnaire-based Data

Potential confounders were identified *a priori* based on existing literature (age [22], gender [23], adiposity [2], smoking status [5], socioeconomic position [4] and health status [24], [25]). Anthropometric measures were taken at clinic or nurse home visits. Standing height was measured to the nearest mm using a stadiometer and weight was measured in kilograms (Kg) using standardised scales. Body Mass Index (BMI) was calculated as weight divided by height^2^ (kg/m^2^) and was used as a measure of adiposity. Smoking behaviour was assessed by self-completed questionnaire (CaPS) or medical interview (HAS, LBC1921 and NSHD). Smoking status was classified into never, past or current. In CaPS, HAS and NSHD, socioeconomic position was defined by the British Registrar General’s classification of occupation and based on own occupation in adult life. In LBC1921, occupational social class was assessed using the standard UK Classification of Occupations, placing occupation into five categories [11]. This was based on their own highest ranked occupation, or for married women their husband’s. In CaPS, HAS and NSHD, lower socioeconomic position was classified as manual (skilled manual, semi-skilled manual and unskilled) and in LBC1921, it was classified as the three non-professional classes. For health status, reported history of cardiovascular disease and diabetes was available in all of the cohorts and reported history of cancer was available in CaPS, LBC1921 and NSHD. Additional health status data was available in LBC1921 (cerebrovascular disease and dementia) and in NSHD (epilepsy). Poor health status was defined as having one or more of the specified health conditions.

### Statistical Analysis

We used linear regression models to analyse walking speed, chair rise speed and grip strength and logistic regression analysis for standing balance. To take account of protocol variability in blood storage, DNA extraction and measurement of telomere length, we converted the absolute measures to study-specific z-scores. We adjusted the final model for all potential confounders (age, gender, BMI, smoking status, socioeconomic position and health status).

We undertook a series of analyses: (a) cross-sectional analyses between telomere length and physical performance at time 1, (b) cross-sectional analyses between telomere length and physical performance at time 2, (c) prospective analyses between telomere length at time 1 and physical performance at time 2, (d) analyses of change in telomere length in relation to physical performance at time 2 (this examined physical performance at time 2 with telomere length at time 2 conditional on time 1) and (e) analyses of telomere length at time 1 in relation to longitudinal changes in physical performance. This last analysis regressed physical performance at time 2 on telomere length at time 1 and adjusted for physical performance at time 1. Standing balance times were highly skewed and change in standing balance was only possible in NSHD. To identify change in standing balance time we derived a three-level ordinal variable, with the inability to balance for more than or equal to <5 s as the criteria for poor balance. Those with good balance (i.e. times ≥5 s) at times 1 and 2 were used as the baseline group and coded as 0, those who ‘improved’ (i.e. had poor balance at time 1 but not at time 2) (n = 38) were also included in this category, those whose performance declined (i.e. had good balance at time 1 but poor balance at time 2) were coded as 1 and those with consistently poor balance were coded as 2. We then undertook ordered logistic regression using this outcome variable.

We undertook a two stage meta-analysis of individual participant data with each model initially run within each cohort (the first stage). The cohort-specific effect estimates and standard errors were then pooled using meta-analysis (the second stage). We performed random-effects meta-analyses using the DerSimonian and Laird method [26] as our *a priori* was that there would be heterogeneity due to differences in measurement protocols. In model A we adjusted for age and gender and in model B additionally for BMI, smoking status, socioeconomic position and health status. We investigated between study heterogeneity using the I^2^ statistic [27]. We plotted Forest plots in the order of mean age and re-ran meta-analyses with each study taken out in turn to see whether this influenced the I^2^ statistic.

We undertook a series of sensitivity analyses: a) excluding any cohorts which were outliers, b) modelling telomere length in three equal groups (tertiles), c) inclusion of subjects unable to perform the physical performance tests who are initially excluded due to missing outcome data, d) computing sex-specific z-scores for walking speed and chair rise speed, e) using less than 5 s as the criterion for poor balance, f) using the TUG test rather than the 3 m walk test for HAS and g) using the continuous standing balance time score with eyes closed in the analysis for change in balance time for NSHD (Appendix S4).

## Results

Descriptive characteristics of the four studies are shown in Table 1. Age range was from 53 to 80 years at time 1, the youngest cohort being NSHD and the oldest LBC1921. The median time lag and inter-quartile range between times 1 and 2 ranged from 7.5 (7.3–7.8) years in LBC1921 to 10.2 (9.3–10.8) years in NSHD. Variation in mean walking speeds between cohorts was partly due to the difference between studies in the protocols for this test.

**Table 1:** Characteristics of the participants at time 1 and time 2, by study.

Variable	CaPS	HAS	LBC1921	NSHD
N†	966	656	493	2558
Gender (% male)	100	69.4	48.8	46.7
Age at time 1 (years)	64.5 (4.1)	67.0 (2.2)	79.1 (0.6)	53.4 (0.2)
Age at time 2 (years)	72.8 (3.9)	76.3 (2.2)	86.6 (0.4)	62.7 (1.0)
BMI (Kg/m^2^)	27.3 (3.6)	26.9 (3.6)	25.9 (4.1)	26.9 (4.1)
Current smoker (%)	17.6	8.0	2.4	17.6
Lower SEP (%)	59.5	46.2	36.0	22.6
Lower Health Status (%)	47.5	27.1	35.2	8.6
Telomere length at time 1 (kb)	4.3 (1.6)	5.2 (1.6)	4.1 (0.4)	5.6 (1.9)
Telomere length at time 2 (kb)	3.3 (1.3)	3.9 (1.3)	4.2 (0.6)	4.3 (1.3)
Walking speed^a^ (m/min)	–	51.3 (9.9)	66.6 (24.1)	–
TUG speed^b^ (m/min)	35.7 (7.4)	–	–	42.3 (8.4)
Standing balance^c^ (s)	15.7 (4.8–30)	10.3 (4.0–27.6)	–	30 (12.3–30)
cut-point (bottom 20%) (s)	3.8	3.0	–	9.1
Chair rise speed^d^ (stands/min)	–	16.1 (4.4)	–	25.6 (7.4)
Grip strength (Kg)				
Males	–	39.1 (7.9)	28.6 (7.3)	47.1 (11.7)
Females	–	24.4 (7.0)	15.4 (4.0)	27.4 (7.5)

† N is the maximum sample size in age and sex adjusted analyses at either time 1 or time 2. Results are presented as mean (SD), unless otherwise stated and are based on complete case analysis. Physical performance measures are taken from time 1. Covariates are given at time 1 unless otherwise stated. Health status was at time 2 in CaPS.

a Walking speed in HAS is based on a 3 m walk at normal page and in LBC1921 it is a 6 m walk undertaken as quickly but as safely as possible.

b Walking speed in CaPS and NSHD is based on the get up and go test (TUG), which involves walking 6 m at a normal pace.

c Standing balance in CaPS, HAS and NSHD is a one-legged stand for 30 s. Median balance times (and inter-quartile range) and cut-point times for bottom 20% are given.

d Chair rise speed in stands/minute is calculated as the number of stands (5 in HAS and 10 in NSHD)/time taken for those stands.

### Meta-analyses

**Figure 1:**
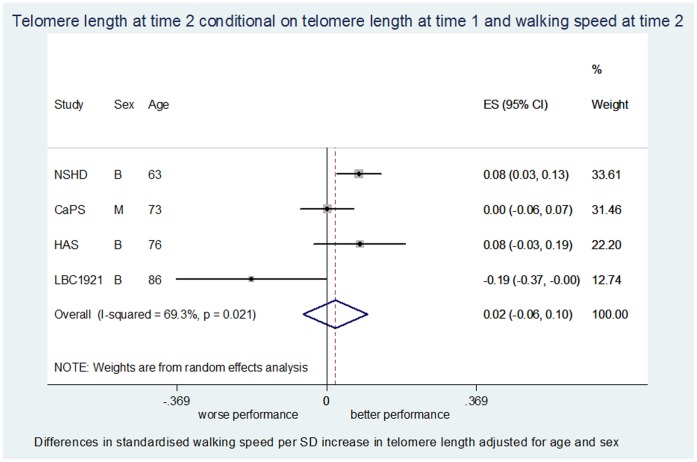
Meta-analysis for the association between telomere length at time 2 conditional on telomere length at time 1 and walking speed at time 2 adjusted for age and sex.

**Figure 2:**
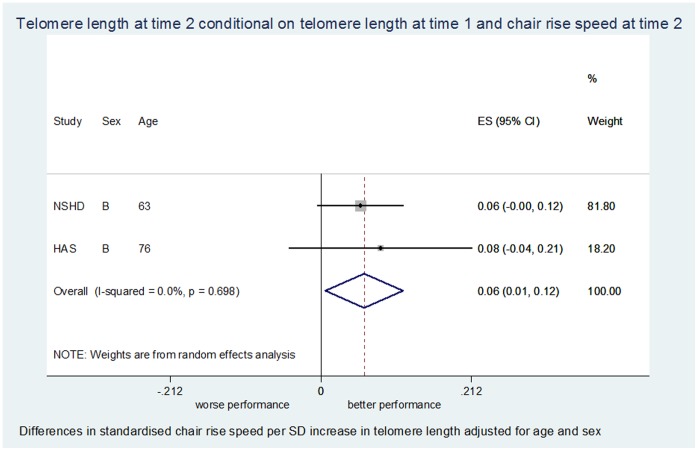
Meta-analysis for the association between telomere length at time 2 conditional on telomere length at time 1 and chair rise speed at time 2 adjusted for age and sex.

Total sample size in the age and sex-adjusted models varied between 1,217 and 3,707 depending on the meta-analysis (Table 2). Associations between telomere length and physical performance from meta-analyses are detailed in Table 2, Figure 1, Figure 2 and Appendix S5.

**Table 2:** Overall summary estimates of effect for the associations between telomere length (TL) at times 1 and 2 and physical performance (PP) at times 1 and 2 from random effects meta-analyses.

			Model A						Model B		
Outcome (PP) and			(Age, sex adjusted)						(Fully adjusted‡‡)		
telomere length (T)**	No*	β†	95% CI	*P* -value	I^2^	*P-* value‡	β†	95% CI	*P-* value	I^2^	*P* -value‡
**Walking speed** †† **(sd score)**											
T1-PP1 (n = 491)	1	0.019	−0.075, 0.112	0.70			0.020	−0.075, 0.116	0.67		
T2-PP2 (n = 2319)	4	0.013	−0.060, 0.086	0.73	66.8%	0.03	0.027	−0.037, 0.091	0.41	56.8%	0.07
T1-PP2 (n = 2907)	4	0.016	−0.033, 0.065	0.52	36.0%	0.20	0.011	−0.031, 0.053	0.61	18.2%	0.30
T2T1-PP2 (n = 2021)	4	0.022	−0.058, 0.101	0.60	69.3%	0.02	0.026	−0.047, 0.099	0.48	62.7%	0.05
T1-ΔPP (n = 144)	1	0.121	−0.021, 0.263	0.09			0.109	−0.043, 0.260	0.16		
**Chair rise speed (sd score)**											
T1-PP1 (n = 2447)	1	0.041	0.001, 0.080	0.04			0.046	0.006, 0.085	0.02		
T2-PP2 (n = 1236)	2	0.052	−0.003, 0.106	0.06	0.0%	0.72	0.042	−0.012, 0.097	0.13	0.0%	0.96
T1-PP2 (n = 2025)	2	0.004	−0.039, 0.047	0.87	0.0%	0.92	0.008	−0.035, 0.051	0.71	0.0%	0.80
T2T1-PP2 (n = 1217)	2	0.061	0.006, 0.115	0.03	0.0%	0.70	0.049	−0.006, 0.104	0.08	0.0%	0.72
T1-ΔPP (n = 1711)	1	−0.016	−0.059, 0.027	0.47			−0.012	−0.055, 0.030	0.57		
**Odds of poor balance**											
T1-PP1 (n = 2578)	1	0.97	0.87, 1.07	0.53			0.95	0.85, 1.05	0.31		
T2-PP2 (n = 2239)	3	1.01	0.91, 1.11	0.93	0.0%	0.64	1.02	0.91, 1.14	0.79	0.0%	0.80
T1-PP2 (n = 2844)	3	1.03	0.94, 1.13	0.52	0.0%	0.63	1.02	0.92, 1.13	0.69	0.0%	0.61
T2T1-PP2 (n = 1949)	3	1.00	0.90, 1.12	0.99	0.0%	0.41	0.99	0.87, 1.11	0.82	0.0%	0.71
T1-ΔPP (n = 1809)	1	1.09	0.95, 1.25	0.20			1.09	0.95, 1.26	0.21		
**Grip strength (Kg)**											
T1-PP1 (n = 3707)	3	0.176	−0.321, 0.673	0.49	68.4%	0.04	0.391	0.090, 0.693	0.01	0.0%	0.48
T2-PP2 (n = 1386)	3	0.230	−0.403, 0.863	0.48	42.7%	0.18	0.243	−0.450, 0.936	0.49	46.5%	0.16
T1-PP2 (n = 2208)	3	0.099	−0.424, 0.622	0.71	36.2%	0.21	0.201	−0.304, 0.706	0.44	27.2%	0.25
T2T1-PP2 (n = 1362)	3	0.301	−0.383, 0.985	0.39	46.6%	0.15	0.216	−0.286, 0.717	0.40	3.8%	0.35
T1-ΔPP (n = 2152)	3	−0.059	−0.383, 0.265	0.72	0.0%	0.59	0.026	−0.310, 0.361	0.88	0.0%	0.42

n = Sample size in age and sex adjusted analyses;

* Number of studies in analyses;

** T1 and T2 are telomere lengths at times 1 and 2 respectively. PP1 and PP2 are physical performance measures at times 1 and 2 respectively.

†† Walking speed includes TUG measures in CaPS and NSHD. T2T1 is telomere length at time 2 conditional on telomere length at time 1 and is a measure of change in telomere length given the initial level. ΔPP is conditional change in physical performance between time 1 and time 2;

† Mean difference in standardised walking speed; Mean difference in standardised chair rise speed; Odds Ratio of poor balance (lowest 20%). For change in balance in NSHD an ordinal group was created for balance from time 1 to time 2 where good balance was balancing ≥5 s and poor balance for <5 s. Here good-good is the baseline group (0) (including poor-good balance), good-poor (1) and poor-poor (2); Mean difference in grip strength;

‡ *P*-value is obtained from the heterogeneity χ^2^;

‡‡ Fully adjusted model is for age, sex, BMI, smoking status, SEP and health status; Telomere length measures at times 1 and 2 have been z-scored. Random effects meta-analyses were undertaken.

#### Walking speed

No association was observed in cross-sectional analyses at time 1 or time 2 between telomere length and walking speed (Table 2). Furthermore, there was little evidence of an association between either telomere length at time 1 and walking speed at time 2 (Table 2), or between change in telomere length and walking speed at time 2 (Table 2; Figure 1). Longer telomeres at time 1 were weakly associated with less decline in walking speed even after full adjustment (*p* = 0.16; Table 2), although this result was based on just one study (LBC1921).

#### Chair rise speed

Longer telomeres at time 1 were associated with quicker chair rise speed at time 1 (Table 2), although this result was based on one study (NSHD). In cross-sectional associations at time 2, longer telomeres were weakly associated with quicker chair rise speed (*p* = 0.06 in age and sex adjusted models and *p* = 0.13 in the fully adjusted model, Table 2). A smaller decline in telomere length between time 1 and time 2 was associated with a quicker chair rise speed when age and sex adjusted (*p = *0.03; Figure 2), weakly attenuated when fully adjusted (*p* = 0.08). There was no evidence of an association between telomere length at time 1 and chair rise speed at time 2 (Table 2), or between telomere length at time 1 and change in chair rise speed (Table 2).

#### Standing balance

There was no evidence of an association in cross-sectional analyses between telomere length at either time 1 or time 2 and balance (Table 2), nor between telomere length at time 1, or change in telomere length and balance at time 2 (Table 2). Moreover, there was little evidence of an association between telomere length at time 1 and change in balance time (Table 2).

#### Grip strength

Whilst there was little evidence of cross-sectional associations at time 1 between telomere length and grip strength when age and sex adjusted (*p = *0.49), longer telomeres at time 1 were associated with stronger grip strength at time 1 after further adjusting for BMI, smoking status, socioeconomic position and health status (*p = *0.01). This was mainly due to the effect of further adjustments for smoking status and health status in HAS, associations which were initially weakly negative becoming weakly positive, albeit consistent with chance. There was little evidence of an association between telomere length and grip strength in cross-sectional analyses at time 2 (Table 2). There was no evidence of an association between telomere length and grip strength in prospective analyses.

### Heterogeneity

There was evidence of moderate heterogeneity between studies in age and sex-adjusted meta-analyses of telomere length and walking speed (I^2^ = 36.0% to 69.3%) and telomere length and grip strength (I^2^ = 36.2% to 68.4%). Omitting the LBC1921 cohort from the meta-analyses of associations between telomere length at time 1 and walking speed at time 2 (I^2^ = 36.0% to 13.0%) and between telomere length at time 1 and grip strength at time 2 (I^2^ = 36.2% to 0.0%), resulted in a reduction in the level of heterogeneity. Omitting the LBC1921 cohort from associations between telomere length at time 1 and grip strength at time 1 resulted in an increase in the level of heterogeneity (I^2^ = 68.4% to 84.1%). Removal of each study in turn from the remaining meta-analyses did not greatly effect the level of heterogeneity (data not shown).

### Sensitivity Analyses

We found little effect on the meta-analyses for a wide range of sensitivity analyses which did not alter our main conclusions (Appendix S4).

## Discussion

In general there was little evidence that telomere length – or maintenance of telomere length over time – was associated with walking speed, balance or grip strength in either cross-sectional or prospective analyses. Our results show that change (smaller decline) in telomere length was associated with quicker chair rise speed at time 2 (where time period between time 1 and time 2 ranged from 7.5 years to 10.2 years) but this was consistent with chance in the fully adjusted model. There was some modest evidence that longer telomeres were associated with quicker chair rise speed at time 1 and time 2 in the partially adjusted model. There was some evidence that longer telomeres were associated with stronger grip strength at time 1 after full adjustment. There was also weak suggestive evidence that telomere length at time 1 was associated with worsening walking speed in prospective analysis but this was consistent with chance in the fully adjusted model.

Our lack of associations between telomere length and grip strength or walking speed are consistent with the literature [10]–[13], with data from LBC1921 [11] included in our study. We are not aware of any studies which have examined the associations between telomere length and either balance or chair rise speed. We found weak evidence of an association between change in telomere length and faster chair rise speed but this could be due to a type I error. The lack of consistency across our various performance measures may be explained by the fact that they are measuring different underlying components of performance. Grip strength is a measure of upper body isometric strength [28], whilst balance, chair rising and walking require lower body strength, balance, postural and motor control [9]. Walking speed and chair rises are more dynamic measures than balance and additionally require leg muscle power (to generate a force quickly) [29]. As the chair rise test makes more demands on cardiorespiratory function than the other measures this might explain the observed differences.

Oxidative stress shortens telomeres [30] and the oxidative stress hypothesis of ageing proposes that oxygen radicals affect ageing and a wide range of diseases [31]. Oxidative stress is thus expected to affect many aspects of human health, including physical performance measures [12]. Indeed, in LBC1921 (79 year olds) [32] oxidative stress genes were associated with both telomere length and grip strength. Oxidative stress might thus be a common underlying cause. LBC1921 had almost no chronological age variation, which might have facilitated finding an effect of telomere length, though the same is true of the younger NSHD birth cohort. The previous LBC1921 analysis only examined grip strength [32], hence the results might not be generalisable to other age groups or physical performance measures.

Shortening of leukocyte telomeres might be an important measure of cellular ageing [8], [32] but there is little evidence that leukocyte telomere length is a bio-aging marker for physical performance [1], [33]. It has been argued that telomere length should be measured in the tissue relevant for the physical perfomance measure being undertaken [13], for example in muscle cells. Whereas associations have been found between telomere length in leukocytes and skin or synovial cells [34], in peripheral blood mononuclear cells and fibroblast cells [24] and in cerebral cortex and liver cells [35], there was no evidence of an association between telomere length in white blood cells and buccal cells [36].

### Strengths and Limitations

This is the only pooled study which has looked into the association between telomere length and physical performance and includes three unpublished datasets. We have overcome the limitation of previous cross-sectional studies by using repeat measures of telomere length and physical performance to undertake prospective analyses. The individual participant data meta-analysis enabled us to conduct comparable analyses across studies. We adjusted for age, BMI, smoking status and socio-economic position as potential confounders in a standardised fashion but we cannot exclude the possibility of residual confounding. Indeed, some potential confounders including physical fitness (e.g. VO_2_ max) [37], [38] could not be taken into account as they had not been measured in the HALCyon cohorts. A further strength was that we examined four different objective measures of physical performance. Telomere length was measured by the same group for all four cohorts and there were a series of methodological checks. Inter-assay coefficients of variation were measured repeatedly and were always found to be below 6%. Plate-to-plate efficiency variation was below 1% for the reference gene PCR and below 3% for telomere PCR. Furthermore the intra-plate single-well efficiency coefficient of variation was below 3%.

Although we have pooled the data from four cohorts, we still may be underpowered. There was some evidence of moderate heterogeneity between studies. One possible source of heterogeneity is the difference between protocols for assessing physical performance and to overcome such differences we harmonised the measures. Furthermore, with such a small number of studies, there will be large 95% confidence intervals on the I^2^ estimate of heterogeneity. We undertook sensitivity analyses by including participants unable to complete the walking speed, chair rise speed, grip strength or balance tests in the bottom 20% and repeating the analysis but this made little difference to the results.

Telomere length measurements made in the four cohorts used in this study yielded highly consistent and reproducible results. Previously, the high throughput qPCR methodology used in these analyses has been criticised that it is not sufficiently reproducible and insufficiently sensitive. In a direct and blinded comparison with Southern blot methodology, using the same 50 DNA samples, qPCR correlated highly (r values >0.9), but the inter-assay CV measurement for the qPCR was 6.45%, and only 1.74% for Southern blotting [39]. In the present analyses, the inter-assay CV was consistently below 6%, in keeping with the majority of observations in the field made using this methodology. Hence, assay sensitivity and reproducibility do not appear to be major issues in this study.

## Conclusions

Whilst leukocyte telomere length might be a biomarker for cellular ageing, we found only weak evidence that it is a bio-aging marker for physical performance. This is in spite of our relatively large sample size and having repeat measures of telomere length. Whilst we cannot exclude a modest association, we believe that a strong association is unlikely though this may not be true for more specific tissues, such as muscle cells. The use of telomere length for screening or targeting interventions to reduce decline in physical performance is unlikely to be helpful.

## Supporting Information

Appendix S1 **Details of cohorts.**
(DOCX)

Appendix S2 **Table showing the availability of different physical performance measures at baseline (T1) and follow-up (T2).**
(DOCX)

Appendix S3 **Details of primer sequences and DNA isolation.**
(DOCX)

Appendix S4 **Sensitivity analyses.**
(DOCX)

Appendix S5 **Supplementary Figures.**
(DOCX)
