# Supplementary material for: Telomere Length and Physical Performance at Older Ages: An Individual Participant Meta-Analysis
Source: PLoS One. 2013 Jul 26;8(7):e69526. doi: 10.1371/journal.pone.0069526 (PMC3724915; doi:10.1371/journal.pone.0069526)
Supplement: Appendix S1 — Details of cohorts. (DOCX) [file pone.0069526.s001.docx]

**Appendix S1.** Details of cohorts.

*Caerphilly Prospective Study (CaPS)*

CaPS is a cohort of men born between 1920 and 1939 who were recruited between 1979 and 1983 from Caerphilly and adjacent villages in South Wales [1]. 2512 men (with a response rate of 89%) were seen when they were 45-59 years and were followed up at phase 2 (1984-1988), phase 3 (1989-1993), phase 4 (1993-1996) and phase 5 (2002-2004). An additional 447 men of similar age, who had moved into the defined study area, were recruited at phase 2. Telomere length measures at phase 4 (time 1) and phase 5 (time 2) and physical performance measures at phase 5 (time 2) are included in the current study.

*Hertfordshire Ageing Study (HAS)*

HAS is a cohort of women and men born in North Hertfordshire between 1920 and 1930 with detailed birth and infant records available and who were still living there in 1994-1995 (wave 1), aged 63-73 years [2]. A second follow-up of HAS was carried out in 2003-2005 (wave 2). Telomere length and physical performance measures at wave 1 (time 1) and wave 2 (time 2) are included in the current study.

*Lothian Birth Cohort 1921 (LBC1921)*

In 1932 the Scottish Mental Survey administered a validated cognitive ability test to 11 year old school pupils (born in 1921) across Scotland. In 1999-2001, surviving members of this survey who were still living in the Lothian area were recruited to participate in LBC1921 (wave 1) [3], with 550 participants, with a mean age of 79 years taking part. The LBC1921 cohort has since been followed up at mean ages of 83 (wave 2), 87 (wave 3) and 90 (wave 4). Telomere length and physical performance measures at wave 1 (time 1) and wave 3 (time 2) are included in the current study.

*MRC National Survey of Health and Development (NSHD)*

NSHD is the 1946 British birth cohort and is a representative sample of people born in England, Scotland and Wales during one week in March 1946 who have been followed up prospectively since birth [4,5]. Telomere length and physical performance measures at age 53 (time 1) and age 60-64 years (time 2) are included in the current study.

**References**

1. Smith GD, Ben-Shlomo Y, Beswick A, Yarnell J, Lightman S, et al. (2005) Cortisol, testosterone, and coronary heart disease: prospective evidence from the Caerphilly study. Circulation 112: 332-340.
2. Syddall HE, Simmonds SJ, Martin HJ, Watson C, Dennison EM, et al. (2010) Cohort profile: The Hertfordshire Ageing Study (HAS). Int J Epidemiol 39: 36-43.
3. Deary IJ, Gow AJ, Pattie A, Starr JM (2011) Cohort Profile: The Lothian Birth Cohorts of 1921 and 1936. Int J Epidemiol 41: 1576-84.
4. Wadsworth M, Kuh D, Richards M, Hardy R (2006) Cohort Profile: The 1946 National Birth Cohort (MRC National Survey of Health and Development). Int J Epidemiol 35: 49-54.
5. Kuh D, Pierce M, Adams J, Deanfield J, Ekelund U, et al. (2011) Cohort Profile: Updating the cohort profile for the MRC National Survey of Health and Development: a new clinic-based data collection for ageing research. Int J Epidemiol 40: e1-9.
