# Supplementary material for: Telomere Length and Physical Performance at Older Ages: An Individual Participant Meta-Analysis
Source: PLoS One. 2013 Jul 26;8(7):e69526. doi: 10.1371/journal.pone.0069526 (PMC3724915; doi:10.1371/journal.pone.0069526)
Supplement: Appendix S2 — Table showing the availability of different physical performance measures at baseline (T1) and follow-up (T2). (DOCX) [file pone.0069526.s002.docx]

**Appendix S2**

**Table S1:** Availability of different physical performance measures at baseline (T1) and follow-up (T2)

| Measure |  | Cohort | | | |
| --- | --- | --- | --- | --- | --- |
|  |  | CaPS | HAS | LBC | NSHD |
| Walking speed | T1 |  |  | X |  |
|  | T2 | X | X | X | X |
| Chair rises | T1 |  |  |  | X |
|  | T2 |  | X |  | X |
| Grip strength | T1 |  | X | X | X |
|  | T2 |  | X | X | X |
| Standing balance | T1 |  |  |  | X |
|  | T2 | X | X |  | X |
