# Supplementary material for: Telomere Length and Physical Performance at Older Ages: An Individual Participant Meta-Analysis
Source: PLoS One. 2013 Jul 26;8(7):e69526. doi: 10.1371/journal.pone.0069526 (PMC3724915; doi:10.1371/journal.pone.0069526)
Supplement: Appendix S3 — Details of primer sequences and DNA isolation. (DOCX) [file pone.0069526.s003.docx]

**Appendix S3.** Primer sequences and DNA Isolation

***Primer Sequences:***

Telomeric Primers used were (HPLC purified):

Tel A 5’-CGGTTTGTTTGGGTTTGGGTTTGGGTTTGGGTTTGGGTT-3’

Tel B 5’-GGCTTGCCTTACCCTTACCCTTACCCTTACCCTTACCCT-3’

Control Gene primers (HPLC purified) for Ribosomal protein 36B4:

36B4-F CAGCAAGTGGGAAGGTGTAATCC

36B4-R CCCATTCTATCATCAACGGGTACAA

All samples were supplied for telomere length measurements at 5ng/ul. As described on the manuscript “To correct for inter-plate variation, four internal control DNA samples of known telomere length were run within each plate and used to generate a regression line by which values of relative telomere length for the actual samples were converted into absolute telomere lengths in base pairs.” These internal controls, whose telomere length in base pairs had been previously assessed by Southern Blot, were as follow: BJ fibroblast Cell line with long telomere length (11.9kb); commercial female human placenta DNA, SIGMA cat # D3035 (3.9kb); HeLa cell line (2.1kb); and pooled PBMC DNAs from 3 donors aged between 21 and 52 years (5.5kb). For each plate, with their values of relative telomere length and their known values of telomere length in base pairs we generated a regression line (r=0.97) that was applied to convert relative telomere length to telomere length in base pairs.

***DNA Isolation***

For all five cohorts, whole EDTA blood DNA was extracted.

**Caerphilly**: The CaPS phase 4 were extracted by standard salting out protocol [6]. Our 'concentrated' non-normalised stocks are made up in TE but normalising dilutions are done with water. Stock DNA quantitation was using picoGreen, read on a fluorimager. Stock DNAs are stored at -80C.

**LBC1921:** DNA extraction was performed by MRC Technology, Western General Hospital, Edinburgh. 2-10ml whole blood was collected into tubes containing sodium-EDTA. Samples were stored at 4˚C for up to 24 hours before processing. White blood cells were isolated from the samples within 24 hours of delivery. Pelleted white blood cells were stored at -20˚C prior to extraction of DNA. Genomic DNA was extracted from the white blood cells using Nucleon BACC2/3 kits according to manufacturer’s instructions. The DNA pellet was air-dried for 10-30 minutes then resuspended in 200-800ul TE (Tris EDTA, pH7.6) and left at 4˚C until the pellet was completely dissolved.

The quality of each DNA was checked by electrophoresis on a 0.8% agarose gel. DNA concentration was determined using Picogreen.

**Hertfordshire**: The method used for extracting the DNA from the whole blood samples is a salting out procedure [1]. In this method the red cells are removed by washing 3 times with erythrocyte lysis buffer and discarded. The presence of 0.15M ammonium salt disrupts the red cells leaving the white cells intact.The white cell pellet is lysed and digested with 10% sodium dodecyl sulphate and the enzyme protease in nuclei lysis buffer and incubated overnight at 37C. The next day sodium chloride is added to the solution to deproteinise by dehydration and precipitation. The precipitate is removed by centrifugation. The supernatant is transferred to another tube and the DNA is precipitated out by adding twice the volume of cold absolute ethanol. The DNA pellet is transferred to a microtube and washed with 70% ethanol. The ethanol is poured off and the DNA pellet left to dry for up to an hour. Tris EDTA is added and the DNA is left to dissolve overnight.

The baseline HAS samples were extracted in 1999. A primary stock and working stock was created. The primary stock was stored at -80C long term and the working stock stored in our -20C freezers. The samples were quantified using the picogreen method. The 2nd stage samples were stored in Newcastle awaiting extraction and were sent to the Human Genetics department Southampton for extraction in January 2010. These were extracted in February 2010. A primary stock and working stock was created. The primary stock stored at -80C and the working stock stored in our -20C freezers. The samples were quantified using the Nanodrop Spectrophotometer.

**NHSD:** DNA was extracted and purified from whole blood using the Puregene DNA Isolation Kit (Flowgen, Leicestershire, UK) according to the manufacturer’s protocol [2]. A total of 5 to 7µg of DNA was treated with *Hinf*I or *Pvu*II in a final volume of 25µl. The *Hinf*I and *Pvu*II fragments were separated using agarose electrophoresis (0.8% agarose and 0.5% agarose respectively).

**References**

1. Miller SA, Dykes DD, Polesky HF (1988) A simple salting out procedure for extracting DNA from human nucleated cells. Nuclei Acids Res 16: 1215.
2. Vinall LE, Fowler JC, Jones AL, Kirkbride HJ, de Bolos C, et al. (2000) Polymorphism of human mucin genes in chest disease: Possible significance of MUC2. Am J Respir Cell Mol Biol 23: 678-686.
