# Supplementary material for: Telomere Length and Physical Performance at Older Ages: An Individual Participant Meta-Analysis
Source: PLoS One. 2013 Jul 26;8(7):e69526. doi: 10.1371/journal.pone.0069526 (PMC3724915; doi:10.1371/journal.pone.0069526)
Supplement: Appendix S4 — Sensitivity analyses. (DOCX) [file pone.0069526.s004.docx]

**Appendix S4.** Sensitivity analyses.

**Methods**

We ran a series of sensitivity analyses:

(1) we repeated meta-analyses for associations of telomere length with walking speed and grip strength excluding LBC1921 because in general it was an outlier.

(2) we modelled telomere length in tertiles to examine for departures from linearity and for comparison with our main analyses in which telomere length was modelled as a continuous variable.

(3) we repeated the meta-analyses for walking speed, chair rise speed, balance and grip strength by classifying those participants unable to perform the tests for health reasons (e.g. surgery, injury or other health problems), who were therefore excluded from the main analyses as having a random value of physical performance within the bottom 20% of the distribution. This was not done for LBC1921 as information on those unable to perform the tests was not available and in HAS no reason was recorded for missing grip strength data.

(4) for walking speed and chair rise speed, we computed sex-specific z-scores to take into account any variation in the distribution of these outcome measures between sexes within studies.

(5) we re-ran analyses of balance using a cut-point for poor balance of 5s rather than the bottom 20^th^ centile.

(6) we repeated the meta-analyses for walking speed for HAS using the TUG test, rather than the standard 3m walk test.

(7) for NSHD we used the continuous standing balance time score with eyes closed (a more sensitive measure than with eyes open) in the analysis for change in balance time. Firstly, we transformed (log_e_) standing balance times as they were positively skewed. We then regressed (log_e_) standing balance time at time 2 on telomere length at time 1 and adjusted for (log_e_) standing balance time at time 1.

**Results**

Our findings in relation to each of the analyses described above were as follows:

(1) in meta-analyses excluding LBC1921 longer telomeres at time 2 were found to be weakly associated with a) faster walking speed at time 2 (0.04; 95% CI -0.02 to 0.09; *p* = 0.19) and b) better grip strength at time 2 (0.42; 95% CI -0.35 to 1.19; *p* = 0.29). Furthermore, change in telomere length was weakly associated with c) faster walking speed at time 2 (0.05; 95% CI -0.01 to 0.11; *p* = 0.08) and b) better grip strength at time 2 (0.52; 95% CI -0.18 to 1.21; *p* = 0.14). However, these associations are consistent with chance. Excluding LBC1921 from the other meta-analyses made little difference to the results (data not shown).

(2) there was little evidence of departures from linearity when treating telomere length as a categorical variable (data not shown).

(3) including the participants who did not complete the walking speed, chair rise speed, balance or grip strength tests for health reasons made little difference to the results (data not shown).

(4) computing sex-specific z-scores for walking speed and chair rise speed had little effect on the meta-analyses.

(5) dichotomising balance using a cut-point of 5 seconds made little difference to the meta-analyses (data not shown).

(6) using the TUG test results for HAS in meta-analyses of walking speed made little difference (data not shown).

(7) using the continuous standing balance time score with eyes closed in the analysis for change in balance time in NSHD made little difference to the results.
