# Supplementary material for: Telomere Length and Physical Performance at Older Ages: An Individual Participant Meta-Analysis
Source: PLoS One. 2013 Jul 26;8(7):e69526. doi: 10.1371/journal.pone.0069526 (PMC3724915; doi:10.1371/journal.pone.0069526)
Supplement: Appendix S5 — Supplementary Figures. (DOCX) [file pone.0069526.s005.docx]

**Appendix S5.** Supplementary Figures

**Figure S1** Meta-analyses for the associations between telomere length at time 2 and walk speed at time 2 (A) and telomere length at time 1 and walk speed at time 2 (B) adjusted for age and sex

**Figure S2** Meta-analyses for the associations between telomere length at time 2 and chair rise speed at time 2 (A) and telomere length at time 1 and chair rise speed at time 2 (B) adjusted for age and sex

**Figure S3** Meta-analyses for the associations between telomere length at time 2 and balance at time 2 (A), telomere length at time 1 and balance at time 2 (B) and telomere length at time 2 conditional on telomere length at time 1 and balance at time 2 (C) adjusted for age and sex

**Figure S4** Meta-analyses for the associations between telomere length at time 1 and grip strength at time 1 (A), telomere length at time 2 and grip strength at time 2 (B), telomere length at time 1 and grip strength at time 2 (C), telomere length at time 2 conditional on telomere length at time 1 and grip strength at time 2 (D) and telomere length at time 1 and change in grip strength (E) adjusted for age and sex

**Figure S1A**

**Figure S1B**

**Figure S2A**

**Figure S2B**

**Figure S3A**

**Figure S3B**

**Figure S3C**

**Figure S4A**

**Figure S4B**

**Figure S4C**

**Figure S4D**

**Figure S4E**
